# Antioxidative and Anti-Inflammatory Activities of Galloyl Derivatives and Antidiabetic Activities of* Acer ginnala*

**DOI:** 10.1155/2017/6945912

**Published:** 2017-03-02

**Authors:** Kwan Hee Park, Kyu Hyeong Yoon, Jun Yin, Thi Tam Le, Hye Sin Ahn, Seong Hye Yoon, Min Won Lee

**Affiliations:** Laboratory of Pharmacognosy and Natural Product Derived Medicine, College of Pharmacy, Chung-Ang University, Seoul 156-756, Republic of Korea

## Abstract

Chromatographic isolation of the 80% MeOH extract of* Acer ginnala* (AG) yielded seven galloyl derivatives: gallic acid (**1**), ginnalin B (**2**), acertannin (**3**), maplexin D (**4**), maplexin E (**5**), quercetin-3-*O*-(2′′-galloyl)-*α*-L-rhamnopyranoside (**6**), and kaempferol-3-*O*-(2′′-galloyl)-*α*-L-rhamnopyranoside (**7**). This is the first study to report the isolation of compounds** 4** and** 5** from AG. Galloyl derivatives** 3**–**7** exhibited potent radical scavenging activities, with** 5 **and** 7** showing particularly strong inhibitory activities against nitric oxide production in lipopolysaccharides- (LPS-) stimulated RAW264.7 cells. In addition, oral administration of AG extract (500 mg/kg b.w.) improved symptoms of hyperglycemia and blunted the increases in serum GOT/GPT levels in a rat model of streptozotocin-induced diabetes. These results suggest that galloyl derivatives (**1**–**7**) are antioxidant and anti-inflammatory agents and that AG extract has potential as a functional material or novel herbal medicine for treating diabetes mellitus.

## 1. Introduction

Diabetes, one of the deathful diseases, is well known worldwide. According to the report of WHO, the number of people with diabetes in 1980 (108 million) has risen to 422 million in 2014 [1]. It is reported that diabetes will be the 7th leading cause of death in 2030 [2]. Between 2 types of diabetes, type 2 diabetes results from ineffective use of insulin, and the majority of people with diabtes worldwide are this type [3]. Oxidative stress is one of the pathways that could induce diabetes, because the high glucose level could simulate free radical production to make imbalance ROS [4]. And it is reported that inflammation is associated with the development of type 2 diabetes, such as proinflammatory cytokines interleukin-1 beta and tumor necrosis factor-alpha [5]. Thus, the antioxidative and anti-inflammatory activities are important ways to treat diabetes.

The genus* Acer* (Aceraceae), commonly known as maple, is the largest group of deciduous trees and shrubs. This genus comprises approximately 130 species occupying a significant part of the northern hemisphere [6]. In Northeast Asia,* Acer* species have been used in pharmaceutical preparations such as decoctions or teas to lower fever, improve eyesight, and protect the liver. In addition,* Acer* species have been used by the native population of eastern Canada as traditional remedies for treating coughs and eye pain [7]. In the present day,* Acer* species are commonly used in commercial products in North America.

*Acer ginnala* (AG), otherwise known as “amur maple,” is native to northeastern Asia. In Republic of Korea, AG has been used as a traditional folk medicine for the treatment of eye disease, wound healing, and diarrhea [8]. The crystalline tannin acertannin was first discovered in AG [9]; the structure of this tannin was later fully elucidated as 2,6-di-*O*-galloyl-1,5-anhydro-D-glucitol [10]. To date, several gallotannins, flavonoids, and terpenoids have been isolated from AG [11, 12]. Moreover, AG has been reported to demonstrate antioxidative, antibacterial, and antitumor activities [13, 14].

The sap of* Acer* species, that is, maple syrup, is believed to be a suitable sweetener for the management of diabetes mellitus [15]. Recently, AG extract was reported to inhibit rat lens aldose reductase and the formation of advanced glycation products [16]. Furthermore, ginnalin A-C from other* Acer* species [17, 18] has been reported to exhibit antihyperglycemic effects in a sucrose-loaded mouse model of diabetes. However, these previous studies used enzymatic tests or the acute glucose tolerance test, which are not sufficient to fully characterize the antidiabetic activity of AG [19]. The aim of this study was to isolate biological compounds from the leaves of AG and to evaluate the hypoglycemic activity of AG in a rat model of streptozotocin- (STZ-) induced diabetes.

## 2. Materials and Methods

### 2.1. Plant Material

The leaves of AG (1 kg) were collected from the Korea National Arboretum (Pocheon, Republic of Korea) in 2010. A voucher specimen (AG2010) was deposited at the herbarium of the College of Pharmacy, Chung-Ang University (Seoul, Republic of Korea).

### 2.2. General Procedures

Large-scale chromatographic isolation was performed on a Sephadex LH-20 column (10–25 *μ*m, GE Healthcare Bio-Sciences AB, Uppsala, Sweden). Daisogel (40–60 *μ*m, Daiso, Osaka, Japan) was used as the stationary phase on a middle pressure liquid chromatography (MPLC) system (Gilson, Seoul, Republic of Korea). The isolation process was monitored by thin layer chromatography (TLC) using a precoated silica gel 60 F254 plate (Merck, Darmstadt, Germany). Spots were detected under UV radiation (254 nm) and by spraying with FeCl_3_ and 10% H_2_SO_4_ or anisaldehyde-H_2_SO_4_, followed by heating. The compounds from the leaves of AG were identified by 1D (^1^H/^13^C) and 2D (COSY, HSQC, and HMBC) nuclear magnetic resonance (NMR) spectroscopy (Varian, Palo Alto, CA) at Chung-Ang University.

### 2.3. Extraction and Isolation

The leaves (1 kg) of AG were extracted three times with 80% methanol at room temperature. After removing the methanol under vacuum, 322.5 g of extract was dissolved in water. The resultant aqueous solution was filtered through Celite 545 (Duksan Pure Chemicals, Ansan, Republic of Korea). Next, 227.5 g of water-soluble concentrated filtrate was applied to a Sephadex LH-20 column (2 kg, 10 cm × 80 cm) equilibrated with distilled water. The column was eluted with a water-methanol gradient system, yielding four fractions. Subfraction 1 (6.4 g) was subjected to Daisogel ODS (300 g, 3 × 50 cm) and eluted from the MPLC system using a 20–100% methanol gradient (5 mL/min, 280 nm) and yielded ginnalin B (**2**, 25 mg). Subfraction 2 (79.5 g) was subjected to Daisogel ODS (300 g, 3 × 50 cm) and eluted from the MPLC system using a 20–100% methanol gradient (5 mL/min, 280 nm) and yielded gallic acid (**1**, 25 mg) and maplexin E (**5**, 20 mg). Subfraction 4 (43.1 g) was subjected to Daisogel ODS (300 g, 3 × 50 cm) and eluted from the MPLC system using a 20–100% methanol gradient (5 mL/min, 280 nm) and yielded gallic maplexin D (**4**, 20 mg), quercetin-3-O-(2′′-galloyl)-*α*-L-rhamnopyranoside (**6**, 150 mg), and kaempferol-3-O-(2′′-galloyl)-*α*-L-rhamnopyranoside (**7**, 850 mg). Subfraction 3 (100.2 g) was recrystallized, yielding acertannin (**3**, 80 g).

#### 2.3.1. Characterization of Compounds Isolated from AG

*Compound* ***1*** *[3,4,5-Trihydroxybenzoic Acid, Gallic Acid] (S1, 2)*

*Gray Amorphous Powder.* ^1^H-NMR (600 MHz, CD_3_OD): *δ* 7.06 (2H, s, H-2, 6); ^13^C-NMR (150 MHz, CD_3_OD): *δ* 109.02 (C-2, C-6), *δ* 120.59 (C1), *δ* 138.15 (C-4), *δ* 144.95 (C-3, 5), *δ* 169.00 (C-7).

*Compound* ***2*** *[6-Galloyl-1,5-anhydroglucitol, Ginnalin B] (S3, 4)*

*Gray Amorphous Powder.*  ^1^H-NMR (600 MHz, CD_3_OD): *δ* 3.01 (1H, t,* J *= 11.1 Hz, H-1b) *δ* 3.11 (2H, m, H-4, 5) *δ* 3.28 (2H, m, H-1a, 3) *δ* 3.71 (1H, m, H-2) *δ* 4.13 (1H, dd,* J *= 4.5, 11.7 Hz, H-6b) *δ* 4.39 (1H, br d,* J *= 11.7 Hz, H-6a) *δ* 6.93 (2H, s, H-2′, 6′); ^13^C-NMR (150 MHz, CD_3_OD): *δ* 64.40 (C-6), *δ* 69.89 (C-1), *δ* 69.99 (C-4), *δ* 70.39 (C-2), *δ* 78.28 (C-3), *δ* 78.65 (C-5), *δ* 108.98 (C-2′, C-6′), *δ* 119.85 (C1′), *δ* 138.66 (C-4′), *δ* 145.73 (C-3′, 5′), *δ* 166.30 (C-7′).

*Compound* ***3*** *[2,6-Digalloyl-1,5-anhydroglucitol, Acertannin] (S5, 6)*

*Gray Amorphous Powder.*  ^1^H-NMR (600 MHz, CD_3_OD): *δ* 3.34 (1H, t,* J *= 11.1 Hz, H-1b) *δ* 3.53 (2H, m, H-4, 5) *δ* 3.72 (1H, t,* J *= 9.0 Hz, H-3) *δ* 4.10 (1H, dd,* J *= 5.5, 11.1 Hz, H-1a) *δ* 4.38 (1H, dd,* J *= 4.5, 11.7 Hz, H-6b) *δ* 4.54 (1H, br d,* J *= 11.7 Hz, H-6a) *δ* 4.90 (1H, m, H-2) *δ* 7.09 (2H, s, H-2′, 6′) *δ* 7.10 (2H, s, H-2′′, 6′′); ^13^C-NMR (150 MHz, CD_3_OD): *δ* 63.52 (C-6), *δ* 66.53 (C-1), *δ* 70.49 (C-4), *δ* 71.77 (C-2), *δ* 75.50 (C-3), *δ* 78.66 (C-5), *δ* 108.81 (C-2′, C-6′), *δ* 108.95 (C-2′′, 6′′), *δ* 119.71 (C1′), *δ* 119.95 (C-1′′), *δ* 138.45 (C-4′), *δ* 138.58 (C-4′′), *δ* 145.02 (C-3′, 5′), *δ* 145.07 (C-3′′, 5′′), *δ* 166.46 (C-7′), *δ* 167.01 (C-7′′′).

*Compound* ***4*** *[2,4-Digalloyl-1,5-anhydroglucitol, Maplexin D] (S7–10)*

*Gray Amorphous Powder.  *Negative FAB-MS:* m/z* 467 [M-H]−; ^1^H-NMR (600 MHz, CD_3_OD): *δ* 3.33 (1H, t,* J *= 10.8 Hz, H-1b) *δ* 3.34 (1H, dd,* J *= 6, 12 Hz, H-6b) *δ* 3.38 (1H, dd,* J *= 2.4, 12 Hz, H-6a) 3.46 (1H, ddd,* J *= 2.4, 5.4, 9.6 Hz, H-5) *δ* 3.80 (1H, t,* J *= 9.0 Hz, H-3) *δ* 3.98 (1H, dd,* J *= 5.4, 10.8 Hz, H-1a) *δ* 4.80 (2H, m, H-2, 4) *δ* 6.97 (2H, s, H-2′, 6′) *δ* 6.97 (2H, s, H-2′′, 6′′); ^13^C-NMR (150 MHz, CD_3_OD): *δ* 61.23 (C-6), *δ* 66.47 (C-1), *δ* 72.00 (C-4), *δ* 72.30 (C-2), *δ* 72.95 (C-3), *δ* 79.94 (C-5), *δ* 109.23 (C-2′, C-6′), *δ* 109.27 (C-2′′, 6′′), *δ* 119.51 (C1′), *δ* 119.73 (C-1′′), *δ* 138.83 (C-4′), *δ* 138.93 (C-4′′), *δ* 145.81 (C-3′, 5′), *δ* 145.83 (C-3′′, 5′′), *δ* 165.49 (C-7′), *δ* 165.77 (C-7′′).

*Compound* ***5*** *[2,4,6-Trigalloyl-1,5-anhydroglucitol, Maplexin E] (S11–14)*

*Gray Amorphous Powder.  *Negative FAB-MS:* m/z* 619 [M-H]−;  ^1^H-NMR (600 MHz, CD_3_OD): *δ* 3.45 (1H, t,* J *= 11.2 Hz, H-1b) *δ* 3.85 (1H, ddd,* J *= 2.1, 5.1, 9.6 Hz, H-5) *δ* 4.03 (1H, t,* J *= 9.3 Hz, H-3) *δ* 4.18 (1H, dd,* J *= 5.4, 11.2 Hz, H-1a) *δ* 4.21 (1H, dd,* J *= 5.1, 11.7 Hz, H-6b) *δ* 4.42 (1H, dd,* J *= 2.1, 11.7 Hz, H-6a) *δ* 5.02 (1H, ddd,* J *= 5.4, 9.6, 11.2 Hz, H-2) *δ* 5.22 (1H, t,* J *= 9.6 Hz, H-4) *δ* 7.09–7.11 (2H×3, each s, H-2′, 2′′, 2′′′, 6′, 6′′, 6′′′); ^13^C-NMR (150 MHz, CD_3_OD): *δ* 62.7 (C-6), *δ* 66.6 (C-1), *δ* 71.1 (C-4), *δ* 71.8 (C-2), *δ* 73.4 (C-3), *δ* 76.8 (C-5), *δ* 108.9–109.4 (C-2′, 2′′, 2′′′, 6′, 6′′, 6′′′), *δ* 119.6–119.8 (C-1, 1′, 1′′′), *δ* 138.5–138.7 (C-4′, 4′′, 4′′′), *δ* 145.0–145.2 (C-3′, 3′′, 3′′′, 5′, 5′′, 5′′′), *δ* 166.3–166.7 (C-7′, 7′′, 7′′′).

*Compound* ***6*** *[Quercetin-3-O-(2*′′*-galloyl)-α-L-rhamnopyranoside] (S15, 16)*

*Yellow Amorphous Powder.*  ^1^H-NMR (600 MHz, CD_3_OD): *δ* 1.03 (3H, d,* J *= 5.4 Hz, H-6′′) *δ* 3.45–3.48 (2H, m, H-4′′, 5′′) *δ* 4.01 (1H, dd,* J *= 3.6, 9.0 Hz, H-3′′) *δ* 5.49 (1H, d,* J *= 1.5 Hz, H-1′′) *δ* 5.63 (1H, dd,* J *= 1.5, 3.6 Hz, H-2′′) *δ* 6.19 (1H, d,* J *= 1.8 Hz, H-6) *δ* 6.37 (1H, d,* J *= 1.8 Hz, H-8) *δ* 6.94 (1H, d,* J *= 8.4 Hz, H-5′) *δ* 7.17 (2H, s, H-2′′′, 6′′′) *δ* 7.33 (1H, d,* J *= 8.4 Hz, H-5′) *δ* 7.34 (1H, dd,* J *= 2.4, 8.4 Hz, H-6′) *δ* 7.36 (1H, d,* J *= 2.4 Hz, H-2′); ^13^C-NMR (150 MHz, CD_3_OD): *δ* 16.41 (C-6′′), *δ* 69.29 (C-3′′), *δ* 70.79 (C-5′′), *δ* 72.10 (C-2′′), *δ* 72.36 (C-4′′), *δ* 93.35 (C-8), *δ* 98.46 (C-6), *δ* 99.10 (C-1′′), *δ* 104.45 (C-10), *δ* 108.99 (C-2′′′, 6′′′), *δ* 115.10 (C-5′), *δ* 115.51 (C-2′), *δ* 119.81 (C-1′′′), *δ* 121.41 (C-6′), *δ* 121.49 (C-1′), *δ* 134.22 (C-3), *δ* 138.55 (C-4′′′), *δ* 144.99 (C-5′′′), *δ* 145.02 (C-3′′′), *δ* 148.42 (C-4′), *δ* 157.07 (C-9), *δ* 157.88 (C-2), *δ* 161.71 (C-5), *δ* 164.40 (C-7), *δ* 166.06 (C-7′′′), *δ* 177.95 (C-4).

*Compound* ***7*** *[Kaempferol-3-O-(2*′′*-galloyl)-α-L-rhamnopyranoside] (S17, 18)*

*Yellow Amorphous Powder.*  Negative FAB-MS:* m/z* 583 [M-H]−; ^1^H-NMR (600 MHz, CD_3_OD): *δ* 1.02 (3H, d,* J *= 6.0 Hz, H-6′′), *δ* 3.44–3.45 (1H, m, H-5′′), *δ* 3.47 (1H, t,* J *= 9.0 Hz, H-4′′), *δ* 3.99 (1H, dd,* J *= 3.6, 9.0 Hz, H-3′′), *δ* 5.48 (1H, d,* J *= 1.8 Hz, H-1′′), *δ* 5.62 (1H, dd,* J *= 1.8, 3.6 Hz, H-2′′), *δ* 6.19 (1H, d,* J *= 1.8 Hz, H-6), *δ* 6.37 (1H, d,* J *= 1.8 Hz, H-8), *δ* 6.96 (2H, d,* J *= 9.0 Hz, H-2′, 6′), *δ* 7.07 (2H, s, H-2′′′, 6′′′), *δ* 7.79 (2H, d,* J *= 9.0 Hz, H-3′, 5′); ^13^C-NMR (150 MHz, CD_3_OD): *δ* 16.4 (C-6′′), *δ* 69.3 (C-3′′), *δ* 70.8 (C-5′′), *δ* 72.1 (C-2′′), *δ* 72.3 (C-4′′), *δ* 93.4 (C-8), *δ* 98.5 (C-6), *δ* 99.1 (C-1′′), *δ* 104.5 (C-10), *δ* 109.0 (C-2′′′, 6′′′), *δ* 115.2 (C-3′′′, 5′′′), *δ* 119.8 (C-1′′′), *δ* 121.1 (C-1′), *δ* 130.5 (C-2′, 6′), *δ* 134.2 (C-3), *δ* 138.5 (C-4′′′), *δ* 145.0 (C-3′′′, 5′′′), *δ* 157.1 (C-9), *δ* 157.8 (C-2), *δ* 160.2 (C-4′), *δ* 161.7 (C-5), *δ* 164.4 (C-7), *δ* 166.0 (C-7′′′), *δ* 177.9 (C-4).

### 2.4. Measurement of DPPH Radical Scavenging Activity

Each sample was added to a 0.1 mM DPPH solution in absolute ethanol. After mixing gently and incubating for 30 min at room temperature, the optical density of the solution was measured at 540 nm using an ELISA reader (TECAN, Salzburg, Austria). The DPPH radical scavenging activity was calculated as inhibition rate (%) = [1 − (sample optical density/control optical density)] × 100. The IC_50_ value was defined as the concentration at which 50% of the DPPH free radicals were scavenged. Ascorbic acid was used as a positive control.

### 2.5. Measurement of NBT/Superoxide Scavenging Activity

Each sample was added to a substrate mixture containing EDTA (0.05 mM), hypoxanthine (0.2 mM), and nitroblue tetrazolium (NBT, 1 mM) in 50 mM phosphate buffer (pH 7.5). The reaction was then initiated by the addition of xanthine oxidase (1.2 U/*μ*L, Sigma). The NBT/superoxide radical scavenging activity was calculated as inhibition rate (%) = [1 − (sample optical density/control optical density)] × 100. The IC_50_ value was defined as the concentration at which 50% of the NBT/superoxide radicals were scavenged. Allopurinol (Sigma) was used as a positive control.

### 2.6. Measurement of Inhibition of NO Production

RAW264.7 mouse macrophage cells were seeded onto 96-well plates (3 × 10^4^ cells/well) and incubated for 2 h at 37°C in a humidified atmosphere (5% CO_2_). The cells were then incubated in medium containing 1 *μ*g/mL lipopolysaccharide (LPS, Sigma) and the sample of interest. After incubating for an additional 24 h, Griess reagent (0.1% naphthylethylenediamine and 1% sulfanilamide in 5% H_3_PO_4_ solution, Sigma) was mixed with the culture supernatant. The absorbance of the samples was then measured at 540 nm using a microplate reader, and the amount of nitrite in each sample was calculated from a sodium nitrite standard curve. The inhibition rate of NO production was calculated as inhibition rate (%) = [1 − (sample optical density − blank optical density)/(control optical density − blank optical density)] × 100. The IC_50_ value was defined as the concentration at which the nitrite radicals were reduced by 50%. L-NMMA was used as a positive control.

### 2.7. Induction of Diabetes and Experimental Design

Male Sprague-Dawley rats (130–150 g each) were housed in the departmental animal facility for two weeks before the experiment. Environmental conditions included a temperature of 22–24°C, a 12 h day/12 h night cycle, and relative air humidity of 40–60%. The animals were provided with standard rodent diet and water ad libitum and were divided into 4 groups (*n* = 6 per group). For the induction of experimental diabetes, 45 mg/kg b.w. (0.1 M citrate buffer of pH 4.5 in a volume of 1 mL/kg) of STZ (Sigma, USA) was injected intraperitoneally. Mice with a blood glucose level greater than 300 mg/dL were considered to be diabetic and were included in the experiment. The 24 rats were divided into the following 4 groups: Group I, normal control; Group II, diabetic control; Group III, diabetic rats treated daily with AG extract (500 mg/kg b.w.). Group IV, diabetic rats treated daily with** 3** (AT, 100 mg/kg b.w.), respectively.

### 2.8. Biochemical Analysis

Rats were fasted overnight, after which blood samples were collected from the inferior vena cava. Blood glucose levels were detected using a portable blood glucose meter (Roche Diagnostics GmbH, Germany). GOT and GPT levels were determined using commercial kits (Asan Pharmaceutical, Republic of Korea).

### 2.9. Statistical Analysis

The DPPH, NBT/superoxide, and nitrite radical scavenging activities of each compound were analyzed by one-way analysis of variance (ANOVA) followed by the Student-Newman-Keuls (SNK) post hoc test. The body weight, blood glucose, and GOT/GPT levels of the rats were analyzed by the Mann–Whitney *U* test.

## 3. Results and Discussion

### 3.1. Isolation and Structural Identification

**Figure 1:**
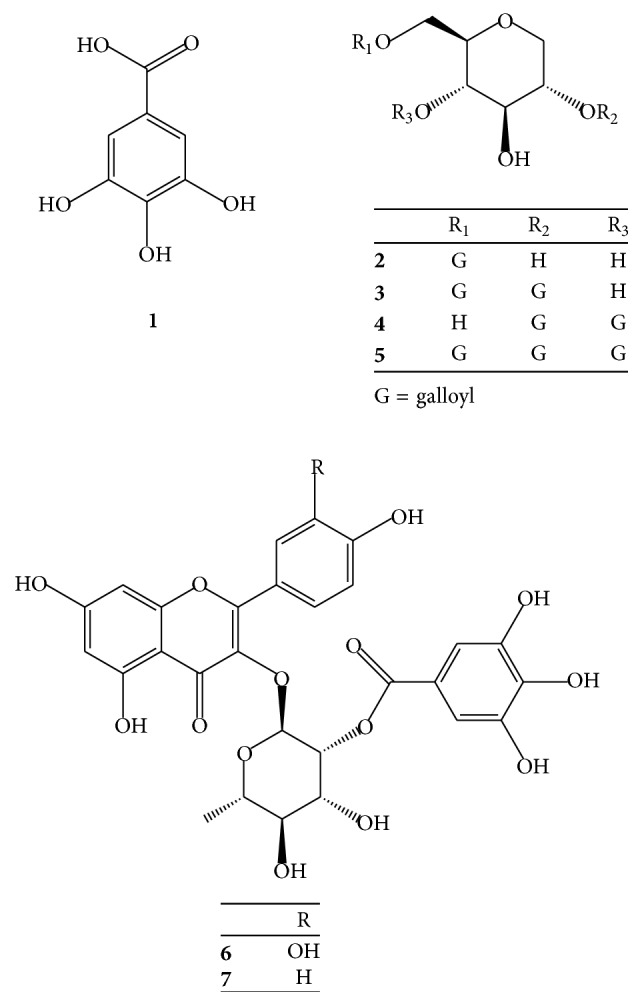
Structures of compounds** 1**–**7** from AG.

The repeated chromatographic isolation of the 80% MeOH extract of AG yielded seven galloyl derivatives: gallic acid (**1**) [8], ginnalin B (**2**) [8, 18], acertannin (**3**) [8, 17], maplexin D (**4**) [20], maplexin E (**5**) [20], quercetin-3-O-(2′′-galloyl)-*α*-L-rhamnopyranoside (**6**) [21], and kaempferol-3-O-(2′′-galloyl)-*α*-L-rhamnopyranoside (**7**) [21] (Figure 1). All isolated compounds were identified by comparing the spectral data with values reported in the literature. This study is the first to report the isolation of compounds** 4** and** 5** from AG.

### 3.2. Evaluation of Antioxidative and Anti-Inflammatory Activities

To assess the antioxidative activities of the compounds isolated from AG, the DPPH radical scavenging ability of each compound was measured. All compounds exhibited potent antioxidative activities, as shown in Table 1. Of particular note, compounds** 3**–**5** exhibited significantly more potent scavenging activity than the positive control (ascorbic acid) (*p* < 0.05). These gallotannins (**3**–**5**) all have more than two galloyl groups, which are known to eliminate free radicals. Moreover, the extent of the DPPH radical scavenging activity depended on the number of galloyl groups (**1**,** 2** <** 3**,** 4**,** 5**). Flavonoids are also well-known natural antioxidants. In particular, flavonoids with a keto-enol moiety at positions C-4 and C-5 and phenolic hydroxyl groups in the B-ring are known to act as crucial electron acceptors. Moreover, compounds** 6** and** 7** possessed an additional galloyl moiety at the O-2 position of the rhamnopyranoside ring. We found that the DPPH radical scavenging activity of compound** 6**, which possessed a 3′,4′-dihydroxyphenyl B-ring, was more potent than that of compound** 7**, which possessed a 4′-hydroxyphenyl B-ring. Moreover, this effect was significantly greater than that of ascorbic acid (*p* < 0.05) (Table 1).

The enzyme xanthine oxidase catalyzes the oxidation of hypoxanthine and xanthine to uric acid, producing a superoxide radical and hydrogen peroxide. Thus, the superoxide radical scavenging activities of the compounds isolated from AG were evaluated using a colorimetric NBT reduction assay. Similar to the DPPH radical scavenging results, compounds** 3**–**5** scavenged superoxide radicals more effectively than compounds** 1** and** 2**, indicating that the number of galloyl groups is crucial for the elimination of superoxide radicals (*p* < 0.05) (Table 1). Flavonoids are also well-known natural xanthine oxidase inhibitors and superoxide radical scavengers. The hydroxyl groups at positions C-5/C-7 and the double bond between positions C-2 and C-3 have both been shown to be essential for effective inhibition of xanthine oxidase, while the hydroxyl group at position C-3′ has been reported to influence superoxide radical scavenging [22]. With respect to these structure-activity relationships, compounds** 6** and** 7** (flavonols) showed either the same or significantly more potent scavenging activity than the positive control, allopurinol (*p* < 0.05) (Table 1). Thus, these results indicate that other bioavailable galloyl derivatives such as oligo-galloyl-substituted acer-tannins or galloyl flavonoids from AG contribute more to the elimination of intracellular oxidative stress compared to compound** 2**.

Next, we confirmed the inhibitory effects of the compounds isolated from AG on nitric oxide production in LPS-stimulated RAW264.7 cells. Macrophages produce various proinflammatory mediators, including the short-lived free radical NO. Moreover, lipopolysaccharide (LPS), a component of the cell wall of Gram-negative bacteria, is one of the most powerful activators of macrophages and stimulates NO production. Of the gallotannins, only compound** 5** (a trigalloyl-substituted tannin) inhibited nitric oxide production; in contrast, treatment with compounds** 1**–**4** did not have any significant effect on NO production. We hypothesize that compounds** 1**–**4** were too hydrophilic to reduce intracellular NO production (Table 1). The *x*log⁡*P* scores of compounds** 1**–**4**, as provided by PubChem, were all negative. Consistent with this tendency, the inhibitory activity of compound** 7**, which possessed a 4′-hydroxyphenyl B-ring, was more potent than that of compound** 6**, which possessed a 3′,4′-dihydroxyphenyl B-ring (*p* < 0.05) (Table 1) [19]. Of particular note, trigalloyl-substituted tannins and kaempferol-derived flavonoids have been reported to reduce iNOS expression via NF-kB inactivation [23, 24].

### 3.3. Evaluation of Antidiabetic Activity

**Figure 2:**
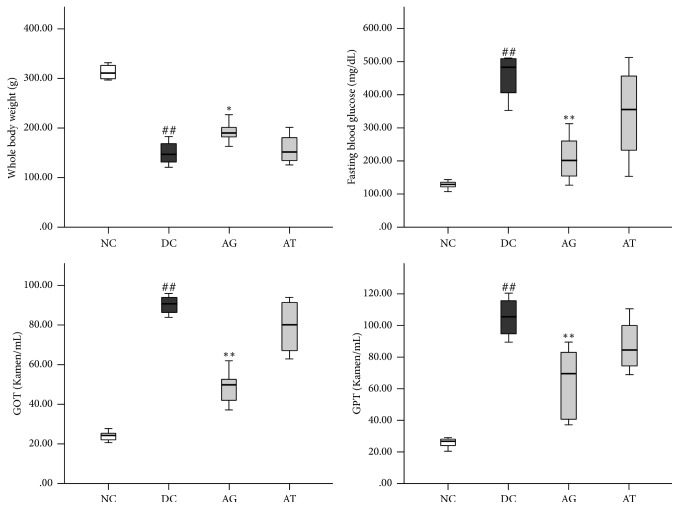
Effects of AG and AT (acertannin) on whole body weight, fasting blood glucose, and GOT and GPT levels in STZ-induced diabetic rats. The experimental diabetic rats were orally given AG (500 mg/kg b.w.) or AT (100 mg/kg b.w.) for 2 weeks (*n* = 6 per group). Results are expressed as median and 75th/25th percentiles (^*∗*^*p* < 0.05 and ^*∗∗*^*p* < 0.01 versus diabetic control group; ^##^*p* < 0.01 versus normal control group).

Streptozotocin is widely applied to induce experimental diabetes due to its ability to selectively target and destroy insulin-producing pancreatic islet beta cells [25]. In the present study, we induced experimental diabetes through IP injection of STZ and evaluated the antidiabetic effect of AG via oral administration of AG extract (500 mg/kg b.w). Alternatively, we administered the major compound of AG, ginnalin A (**3**) (100 mg/kg b.w). The STZ IP injection group exhibited significantly elevated fasting blood glucose and serum GOT/GPT levels compared to the normal control group. Oral administration of AG extract significantly reduced these increases in fasting blood glucose and serum GOT/GPT levels compared to the diabetic control group. However, treatment group of** 3** did not exhibit any significant changes, even though the median values were decreased. In addition, STZ injection resulted in a significant decrease in whole body weight, which was restored by the oral administration of AG. Similar to above, this rescue was not observed in treatment group of** 3** (Figure 2).

Diabetes is a pathological process related to an unbalanced production of ROS, such as hydroxyl radicals (^*∙*^OH), superoxide anions (O_2_), and H_2_O_2_, and RNS such as nitric oxide (NO) and peroxynitrite (ONOO^−^) [26]. Excessive oxidative stress elevates aldose reductase activity and stimulates the hexosamine biosynthetic pathway, as well as increasing formation of advanced glycation end-products [27]. In addition, lipid peroxide production and abnormal immune responses result in various complications such as diabetic retinopathy, nephropathy, neuropathy, and atherosclerosis [28]. Thus, the application of natural antioxidants that offer resistance against oxidative stress to diabetes has been a topic of interest.

STZ has been reported to generate robust production of nitric oxide and hydroxyl radicals or ROS [29], leading to inflammation and apoptosis of *β*-cells in the pancreas and thereby mimicking autoimmune diabetes. Moreover, at high concentrations, acute toxicity in extrapancreatic organs such as the liver has been reported [30]. Oral administration of AG to diabetic rats effectively recovered fasting blood glucose and serum GOT/GPT levels. These results suggest that AG has a protective effect against oxidative stress in STZ-induced diabetic rats.

In contrast, acertannin (**3**), the main crystalline tannin of AG, did not show significant antidiabetic activity. Consistent with this result,** 3** and the monogalloyl- and digalloyl-substituted tannins (**1**,** 2**, and** 4**) did not have any effect on NO production in LPS-stimulated RAW264.7 cells.

*α*-Glucosidase secreted from intestinal chorionic epithelium is a carbohydrate hydrolase. *α*-Glucosidase (EC 3.2.1.20) inhibitors, as a new antidiabetic drug from 1980s, slow down the process of digestion and absorption of carbohydrates by competitively blocking the glucosidase activity. A previous report [31] found that galloyl substitution (more than three substitutions at the 1,5-anhydroglucitol core) resulted in potent inhibition of *α*-glucosidase, with a galloyl group at C-6 being essential for this activity. In particular, the *α*-glucosidase inhibitory activity of** 3** (IC_50_ = 88.42 ± 6.94 *μ*M) was reported to be 10 times less than that of** 5** (IC_50_ = 8.26 ± 0.37 *μ*M) [31]. Moreover, flavonoid hydroxylation and galloylation have been reported to improve the inhibitory activity of *α*-glucosidase [32]. This finding suggests that oligo-galloyl-substituted acer-tannins or galloyl flavonoids from AG also directly influence glucose metabolism. Future studies will aim to elucidate the active constituents of AG and to elucidate the mechanism of action of this extract.

## 4. Conclusion

Chromatographic isolation of the 80% MeOH extract of* Acer ginnala* (AG) yielded seven galloyl derivatives: gallic acid (**1**), ginnalin B (**2**), acertannin (**3**), maplexin D (**4**), maplexin E (**5**), quercetin-3-*O*-(2′′-galloyl)-*α*-L-rhamnopyranoside (**6**), and kaempferol-3-*O*-(2′′-galloyl)-*α*-L-rhamnopyranoside (**7**). Compounds** 3**–**7** exhibited particularly potent radical scavenging activities, and** 5 **and** 7** strongly inhibited nitric oxide production in LPS-stimulated RAW264.7 cells. In addition, oral administration of AG extract (500 mg/kg b.w.) for 2 weeks resulted in significantly lower fasting blood glucose and serum GOT/GPT levels in STZ-induced experimental diabetic rats. These results suggest that the leaves of AG could be useful in the treatment of diabetes mellitus. However, identification of the active constituents of AG responsible for its antidiabetic activity will require further in vivo studies. Such studies could be designed based on reports that acertannin exhibits antidiabetic activity in vitro and in the acute glucose tolerance test in vivo.

## Supplementary Material

The NMR and mass spectrum data of isolated compouds were summited in supplementary material.

## Figures and Tables

**Table 1:** IC_50_ values of compounds **1**–**7** for DPPH scavenging, NBT/superoxide radical scavenging, and inhibition of nitric oxide production by LPS-stimulated RAW264.7 cells.

Compound	IC_50_ (*μ*M)^a^		
	DPPH radical scavenging activity	NBT/superoxide scavenging activity	Inhibition of NO production
**1**	10.44 ± 0.77^b,c^	17.08 ± 1.33^e^	100<
**2**	12.14 ± 0.03^c^	20.80 ± 0.84^e^	100<
**3**	6.87 ± 1.05^a^	2.96 ± 0.14^a^	100<
**4**	6.92 ± 0.52^a^	3.01 ± 0.12^a^	100<
**5**	5.72 ± 0.30^a^	2.83 ± 0.09^a^	36.08 ± 2.12^b^
**6**	12.44 ± 0.30^c^	5.20 ± 0.57^b^	76.46 ± 4.68^c^
**7**	18.92 ± 0.33^d^	12.44 ± 1.54^c,d^	35.62 ± 1.25^b^
Vitamin C	13.11 ± 0.12^c^	—	—
Allopurinol	—	9.75 ± 1.22^c^	—
L-NMMA	—	—	17.23 ± 1.25^a^

^a^Values are presented as mean ± SD (*n* = 3).

Different superscript letters indicate a significant difference (*p* value < 0.05).
