# Supplementary material for: Antioxidative and Anti-Inflammatory Activities of Galloyl Derivatives and Antidiabetic Activities of Acer ginnala
Source: Evid Based Complement Alternat Med. 2017 Mar 2;2017:6945912. doi: 10.1155/2017/6945912 (PMC5352893; doi:10.1155/2017/6945912)
Supplement: Supplementary file 1 — The NMR and mass spectrum data of isolated compouds were summited in supplementary material. [file 6945912.f1.docx]

Supporting data legends

S1. ^1^H-NMR spectrum of **1**

S2. ^13^C-NMR spectrum of **1**

S3. ^1^H-NMR spectrum of **2**

S4. ^13^C-NMR spectrum of **2**

S5. ^1^H-NMR spectrum of **3**

S6. ^13^C-NMR spectrum of **3**

S7. ^1^H-NMR spectrum of **4**

S8. ^13^C-NMR spectrum of **4**

S9. HMBC spectrum of **4**

S10. MS spectrum of **4**

S11. ^1^H-NMR spectrum of **5**

S12. ^13^C-NMR spectrum of **5**

S13. HMBC spectrum of **5**

S14. MS spectrum of **5**

S15. ^1^H-NMR spectrum of **6**

S16. ^13^C-NMR spectrum of **6**

S17. ^1^H-NMR spectrum of **7**

S18. ^13^C-NMR spectrum of **7**

S1. ^1^H-NMR spectrum of **1** (CD_3_OD)


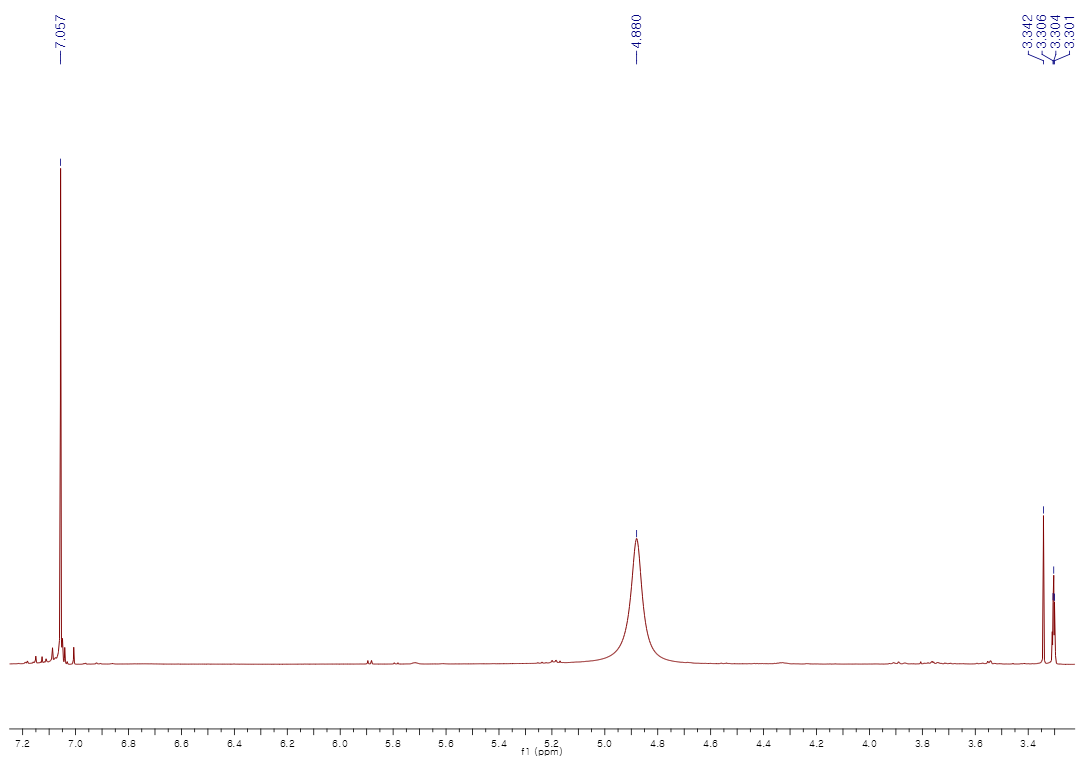


S2. ^13^C-NMR spectrum of **1** (CD_3_OD)

**
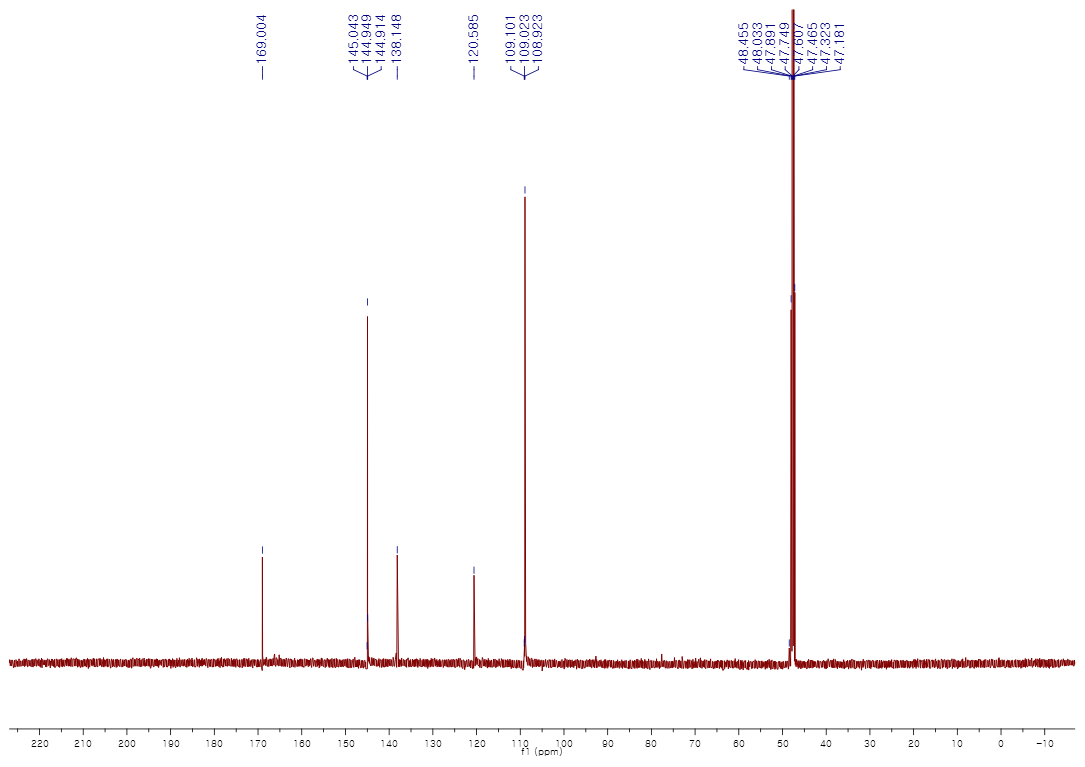
**

S3. ^1^H-NMR spectrum of **2** (CD_3_OD)

**
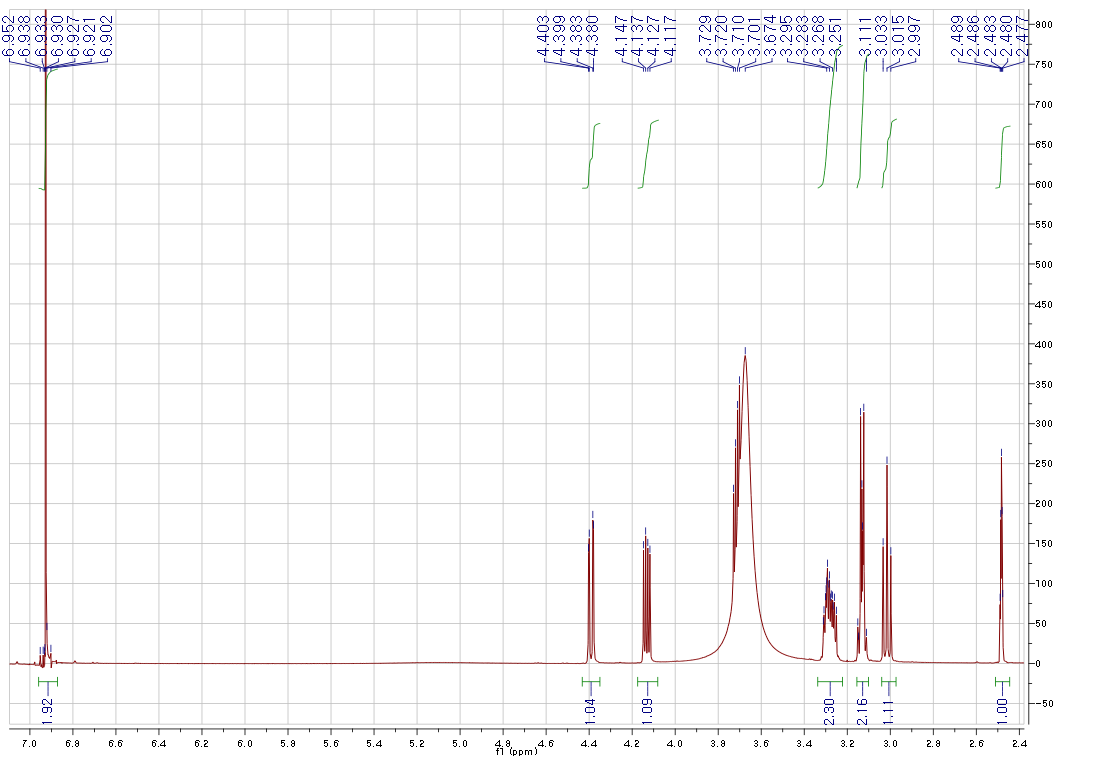
**

S4. ^13^C-NMR spectrum of **2** (CD_3_OD)

**
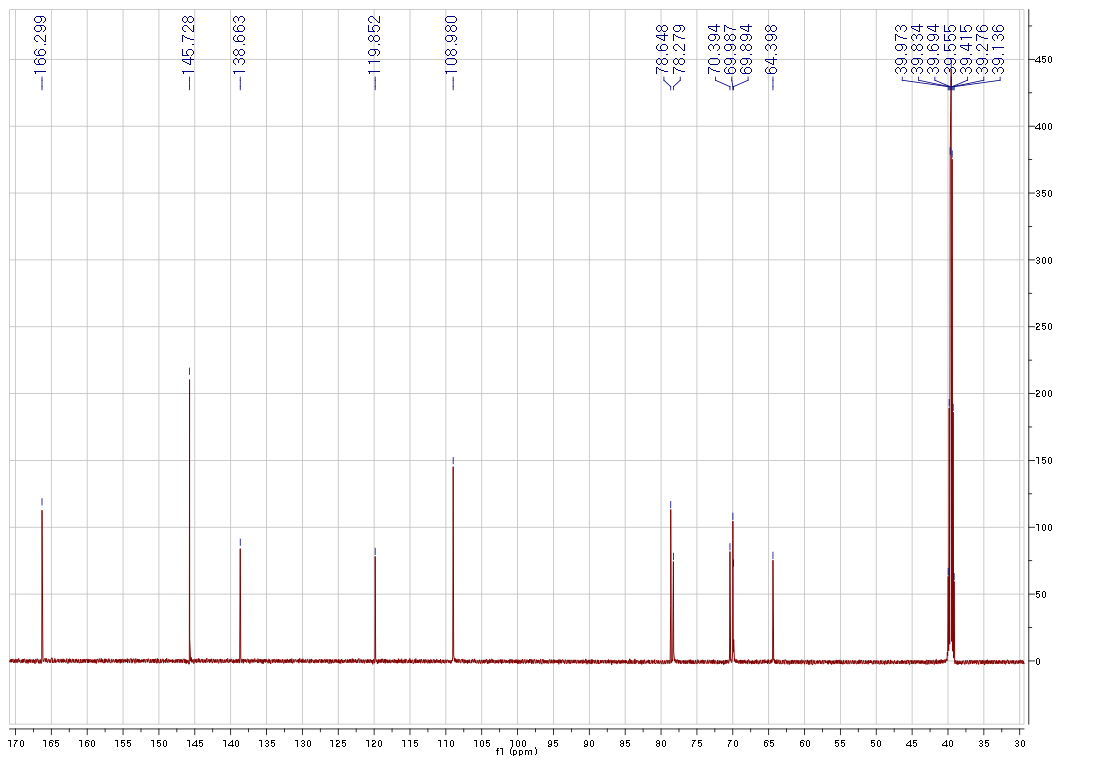
**

S5. ^1^H-NMR spectrum of **3** (CD_3_OD)

S6. ^13^C-NMR spectrum of **3** (CD_3_OD)

S7. ^1^H-NMR spectrum of **4** (CD_3_OD)

S8. ^13^C-NMR spectrum of **4** (CD_3_OD)

S9. HMBC spectrum of **4** (CD_3_OD)


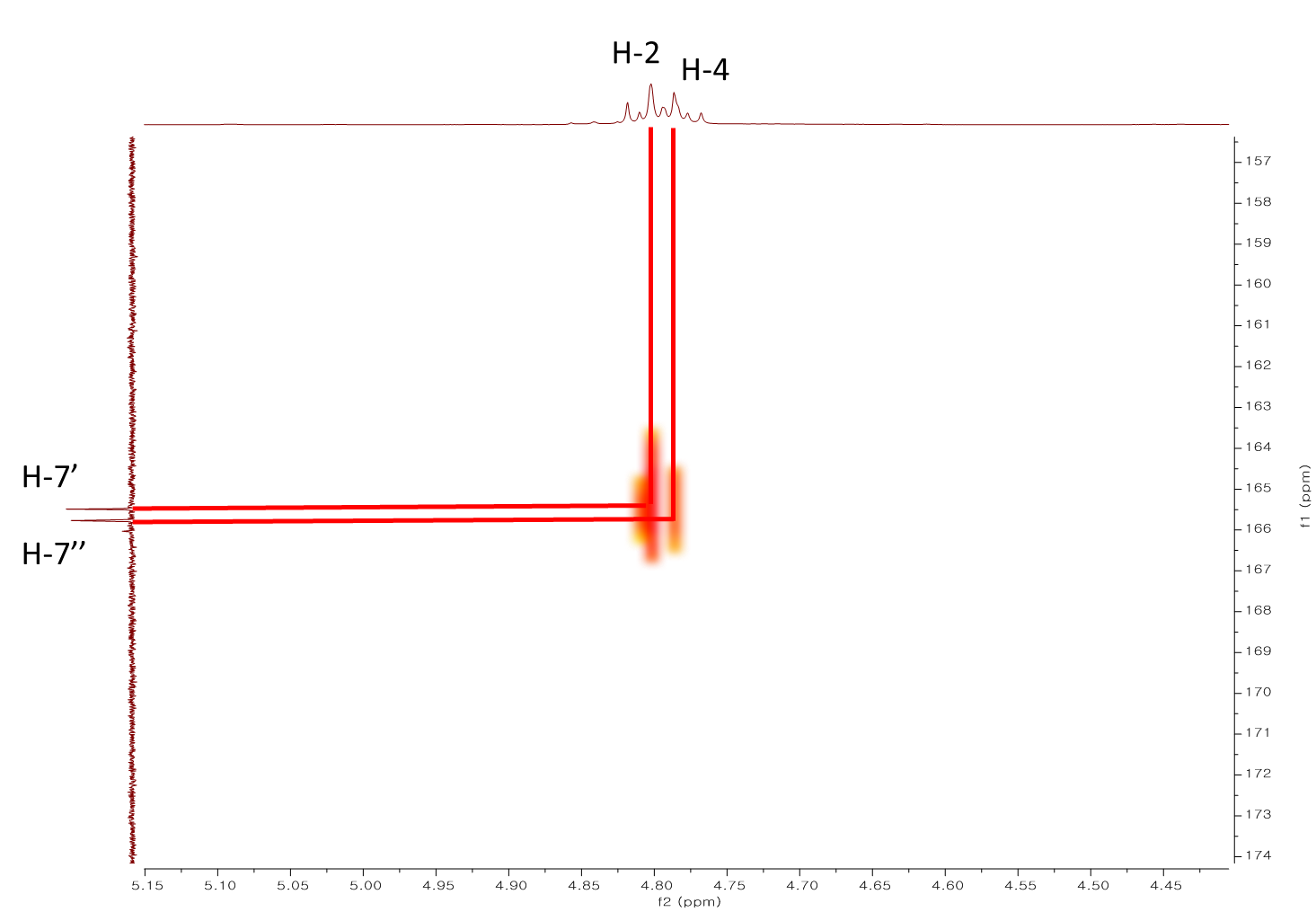


S10. MS spectrum of **4**


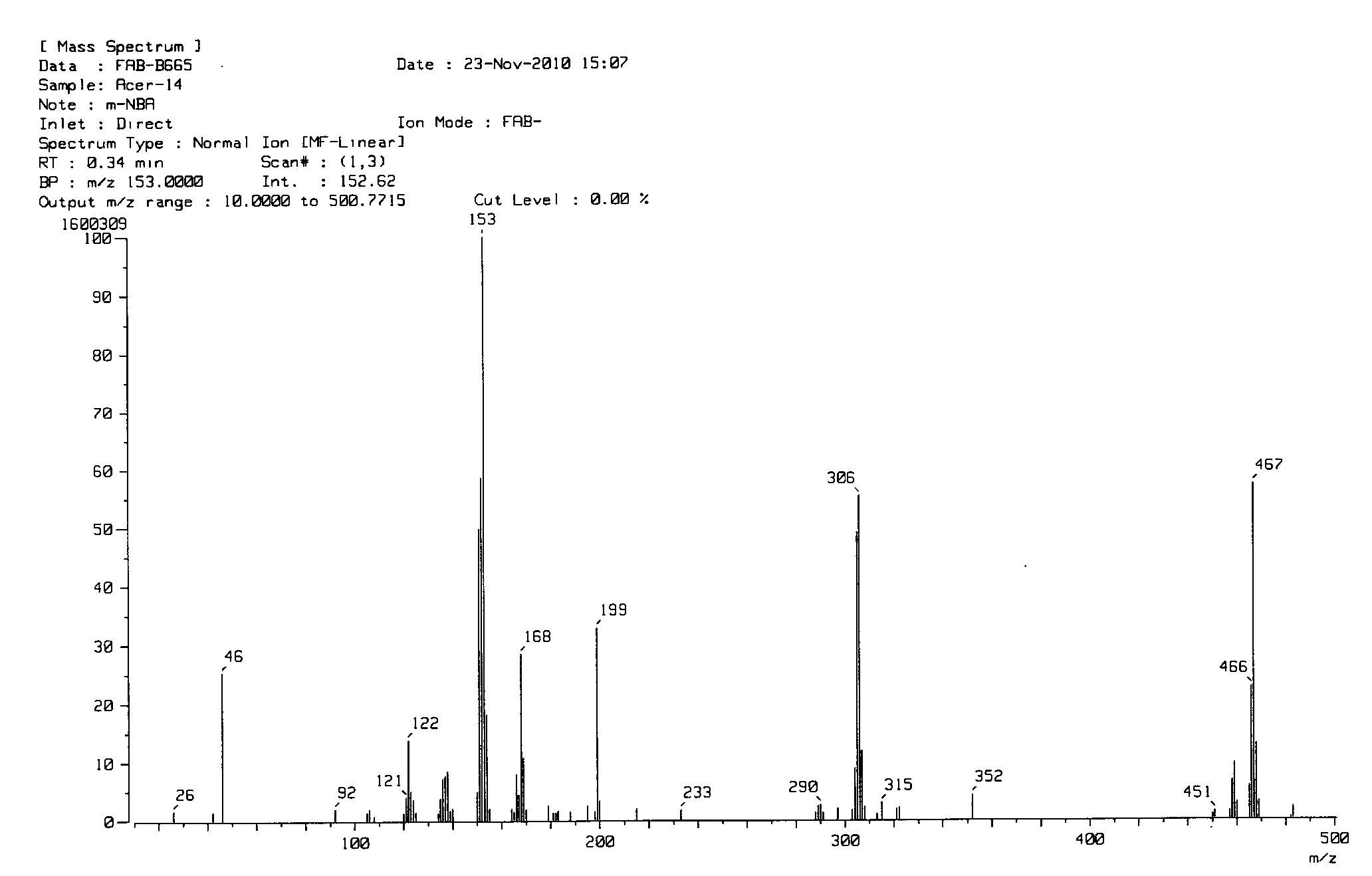


C_20_H_20_O_13_

-Galloyl

S11. ^1^H-NMR spectrum of **5** (CD_3_OD)

**
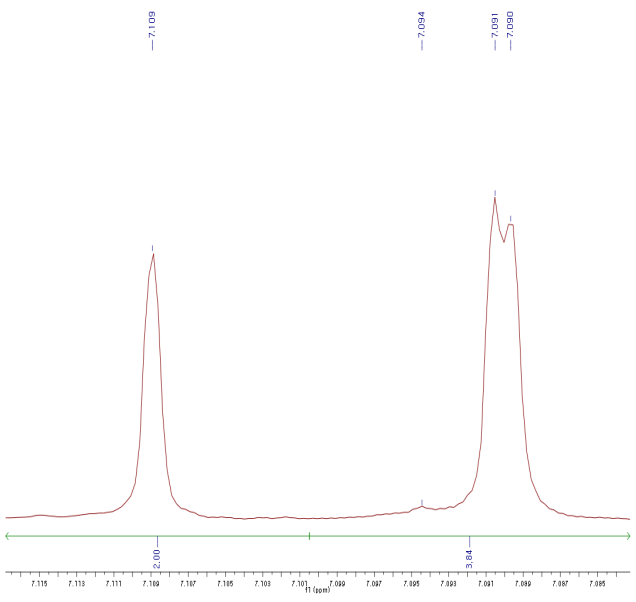
**

S12. ^13^C-NMR spectrum of **5** (CD_3_OD)

**
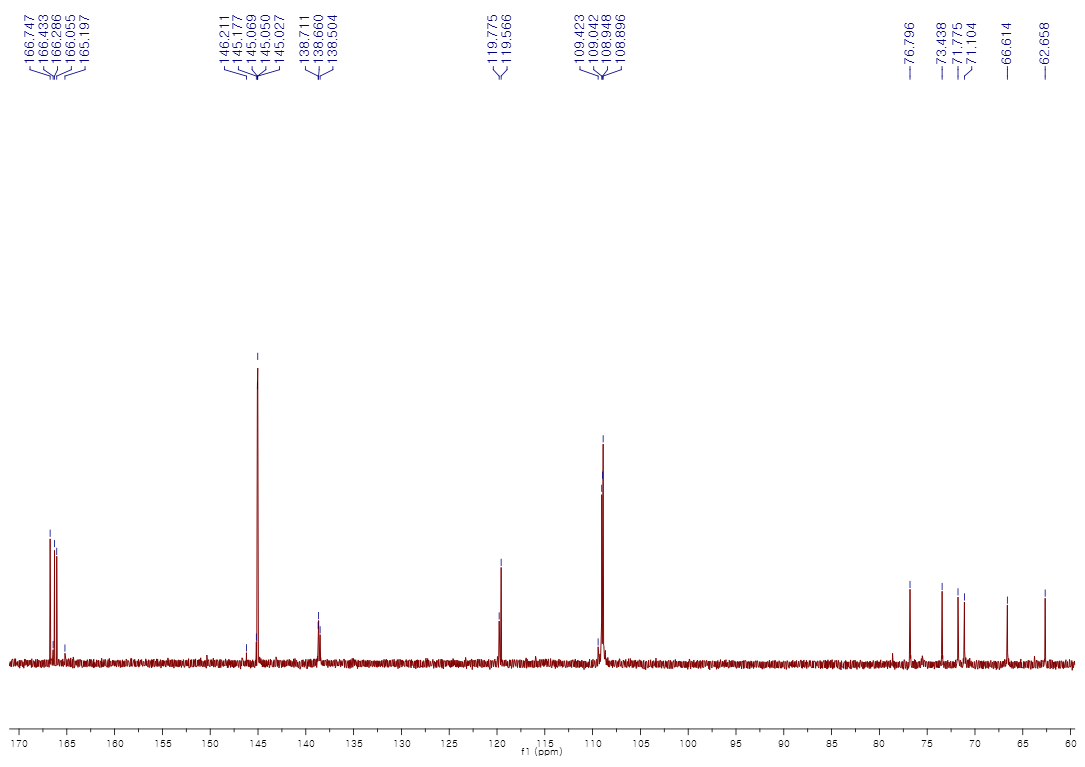
**

S13. HMBC spectrum of **5** (CD_3_OD)

**
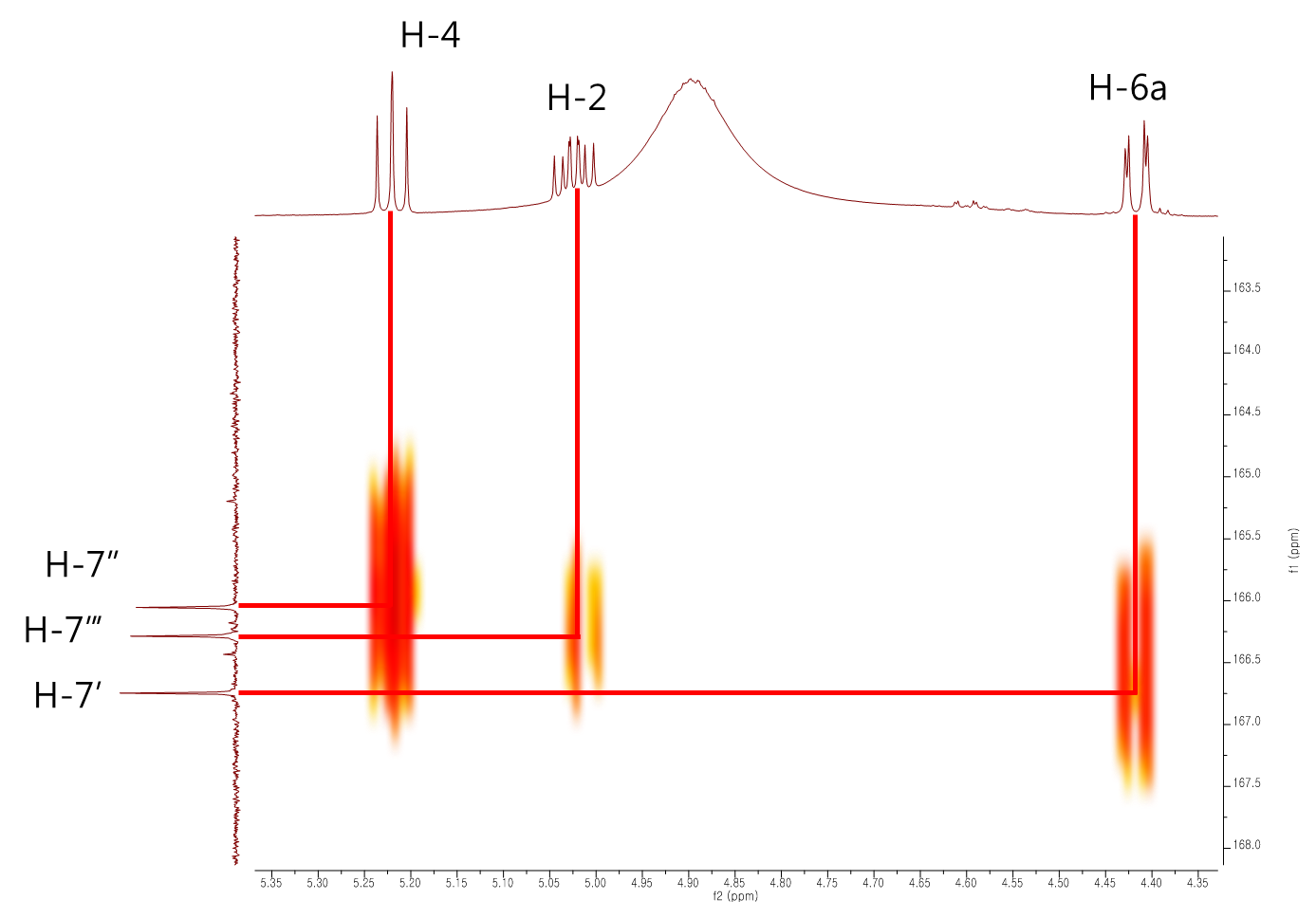
**

S14. MS spectrum of **5**


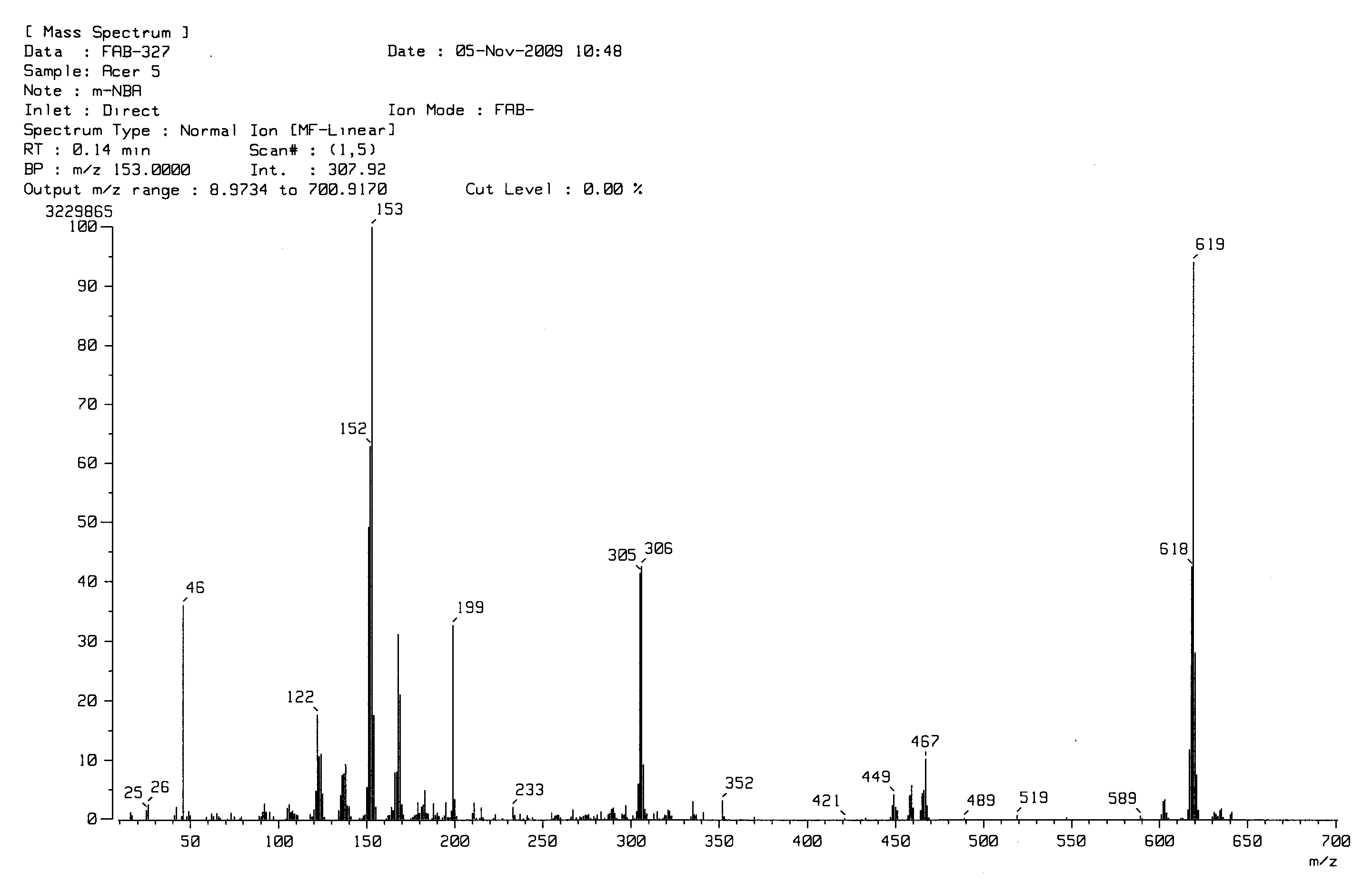


C_27_H_24_O_17_

-Galloyl

S15. ^1^H-NMR spectrum of **6** (CD_3_OD)

S16. ^13^C-NMR spectrum of **6** (CD_3_OD)


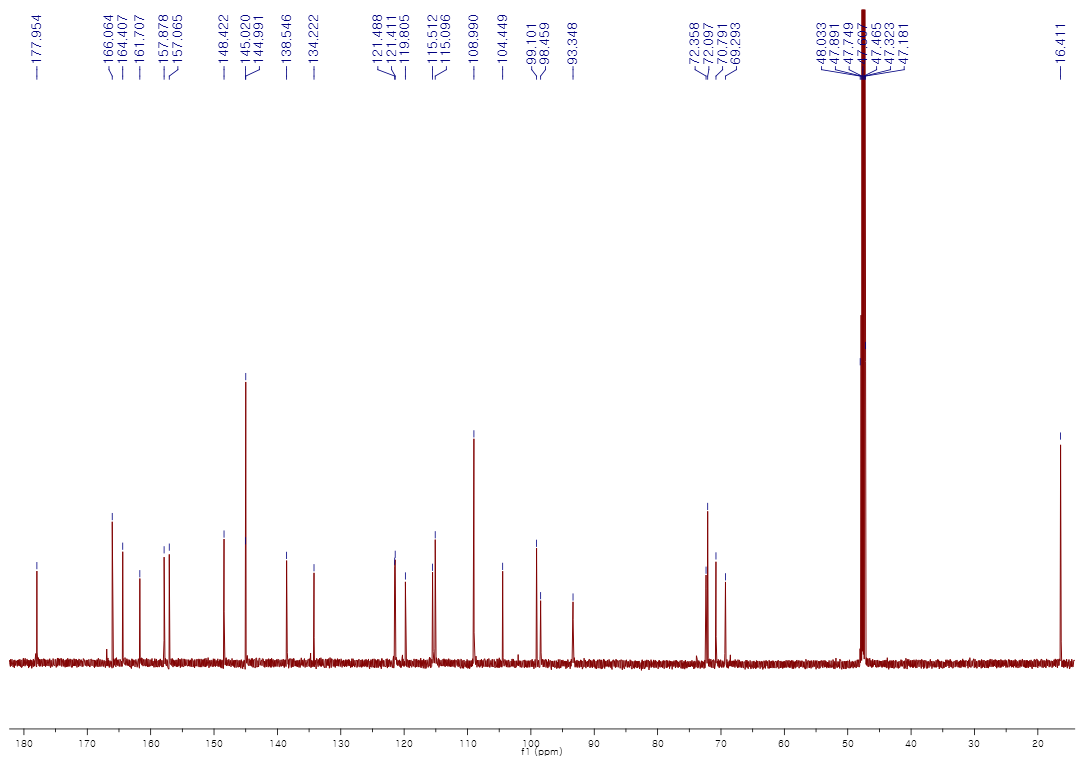


S17. ^1^H-NMR spectrum of **7** (CD_3_OD)

S18. ^13^C-NMR spectrum of **7** (CD_3_OD)

**
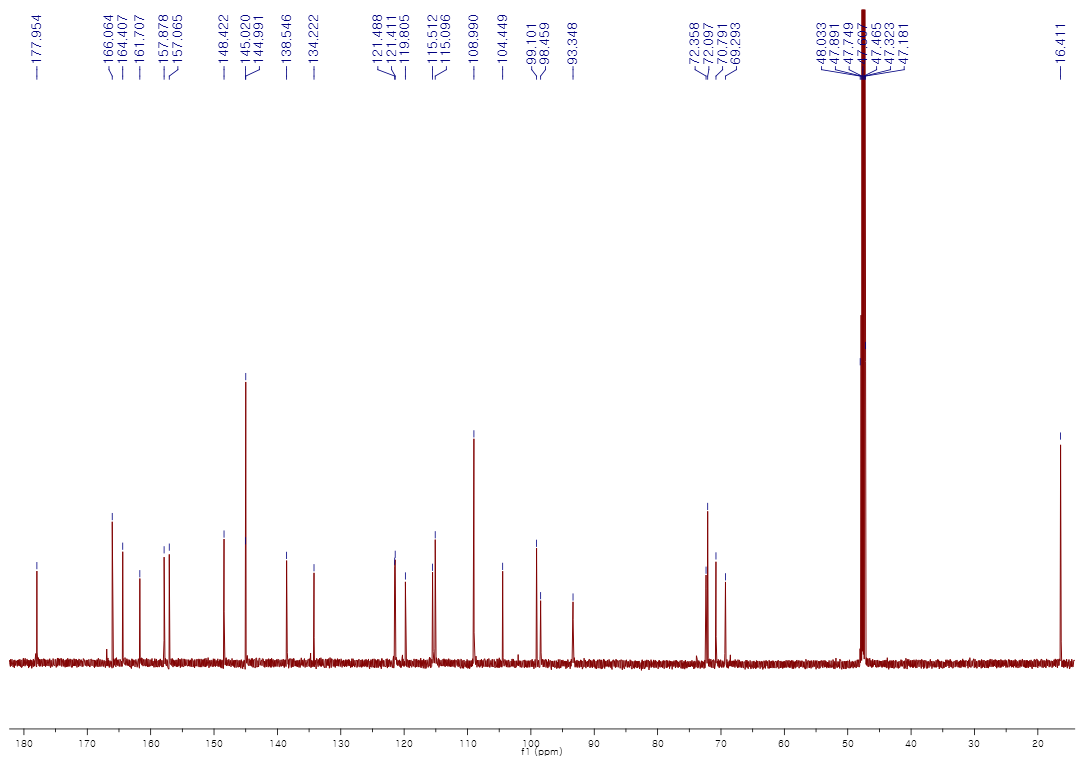
**
